# Staff perceptions of change resulting from participation in a European cancer accreditation programme: a snapshot from eight cancer centres

**DOI:** 10.3332/ecancer.2015.547

**Published:** 2015-06-23

**Authors:** Abinaya Rajan, Anke Wind, Mahasti Saghatchian, Frederique Thonon, Femke Boomsma, Wim H van Harten

**Affiliations:** 1 The Netherlands Cancer Institute, Plesmanlaan 121, Amsterdam 1066CX, The Netherlands; 2 Institut Gustave Roussy, 14 rue Edouard-Vaillant, Villejuif 94805, France; 3 Organisation of European Cancer Institutes (OECI-EEIG) c/o Fondation Universitaire, 11 Rue d’Egmont, Brussels B-1000, Belgium; 4 Integraal Kankercentrum Nederland, Griffeweg 97, Groningen 9723 DV, The Netherlands

**Keywords:** comprehensive health care, quality assurance, health care, quality improvement, accreditation, perception

## Abstract

**Background:**

Healthcare accreditation is considered to be an essential quality improvement tool. However, its effectiveness has been critiqued.

**Methods:**

Twenty-four interviews were conducted with clinicians (five), nurses (six), managers (eight), and basic/translational researchers (five) from eight European cancer centres on changes observed from participating in a European cancer accreditation programme. Data were thematically analysed and verified with participants and checked against auditor’s feedback.

**Results:**

Four change categories emerged: (i) the growing importance of the nursing and supportive care field (role change). Nurses gained more autonomy/clarity on their daily duties. Importance was given to the hiring and training of supportive care personnel (ii) critical thinking on data integration (strategic change). Managers gained insight on how to integrate institutional level data (iii) improved processes within multidisciplinary team (MDT) meetings (procedural change). Clinical staff experienced improved communication between MDTs (iv) building trust (organisational change). Accreditation improved the centre’s credibility with its own staff and externally with funders and patients. No motivational changes were perceived. Researchers perceived no changes. The auditor’s feedback included changes in 13 areas: translational research, biobanks, clinical trials, patient privacy and satisfaction, cancer registries, clinical practice guidelines, patient education, screening, primary prevention, role of nurses, MDT, supportive care, and data integration. However, our study revealed that staff perceived changes only in the last four areas.

**Conclusion:**

Staff perceived changes in data integration, nursing and supportive care, and in certain clinical aspects. Accreditation programmes must pay attention to the needs of different stakeholder groups, track changes, and observe how/why change happens.

## Introduction

Accreditation programmes seem to have become an influential mechanism for improving the performance of many Healthcare organisations (HCOs) [1]. However, the value of accreditation for HCOs still remains unclear [2] especially in terms of clinical outcome improvement [3]. One way of assessing the value of accreditation programmes is to look into the changes that they bring about in HCOs since continuous quality improvement is about culture change [4]. Our study looks at staff perceptions on changes (from eight European cancer centres) resulting from their participation in a European cancer accreditation and designation programme developed by the Organisation of European Cancer Institutes (OECI). Previously, we published an article on the quantitative data gathered by this programme [5]. Here, we present staff perceptions of perceived changes in their cancer centres brought about by the implementation of the qualitative standards.

## Methods

The methods section has been summarised in Table 1.

## Results

**Figure 1.**
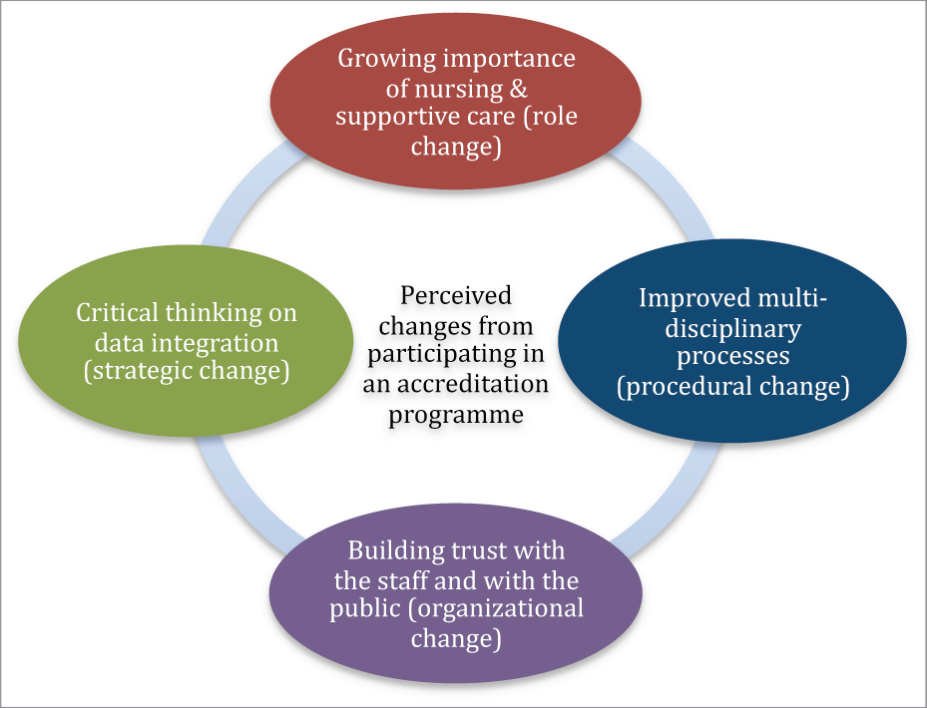
Perceptions of change by staff from cancer centres from participation in a European accreditation programme.

The thematic analysis of the interview data led to four categories (also see Figure 1).

### (i) Growing importance of nursing and supportive care field (role change)

In most centres, psychosocial counselling referrals are initiated by a physician based on his/her knowledge, experience, and attitude to psychosocial counselling. The referral was a solo activity of the doctor and so was not based on a structural multi-disciplinary approach. The number of nurses available per patient was found to be insufficient. The auditors found that some supportive care disciplines only had one representative staff member e.g. one psychiatrist and one social worker. In some cases patients were transferred to other general hospitals to provide supportive palliative care. Workers knew about the palliative and terminal care services outside the centre but the availability of those services was not clearly communicated to patients and was therefore unknown to patients. A job description with specific roles and responsibilities of the functions of supportive care staff was not structurally implemented.

Based on the interviews with nurses, their autonomy seems to have changed quite a lot with regards to their role especially in pain management and palliative care. The clinicians and managers from the majority of centres said that they were expanding their supportive care team. The information flow to those patients on external palliative care seems to have been improved. Staff perceived that allocating more responsibilities to nurses and/or the supportive care team improved because of data integration including access to patient data, and the improved communication within the multidisciplinary team(s) (MDT) of which nursing and supportive care members are also a part.

*‘We have managed to merge certain tasks of physicians and nurses to give additional responsibilities to nurses’.* (Clinician, Centre 6, audited)

*‘Nurses now also report on problems of different processes, programmes for audit of nurses have improved. Now we nurses think critically, now we propose solutions to problems proposed by our unit’*. (Nurse, Centre 4, audited)

*‘Before starting the accreditation process you couldn’t see how accreditation could impact on nurse’s work, but since the start of the process you are saying it’s good to know how you can perform better, self-reflection of nurses has improved’.* (Nurse, Centre 2, unaudited)

*‘There are differences in pain management of patients across Europe. This needs to be addressed and has started to be addressed’.* (Nurse, Centre 6, audited)

*‘We are looking for more social workers, staff for psychological and supportive care’*. (Clinician, Centre 4, after audit and Manager Centre 7, audited)

### (ii) Critical thinking on data integration (strategic change)

In all cancer centres except one which underwent the audit, reviewers felt that it was unclear whether and how an annual report from different departments of the oncology centre was being integrated, and how it helped to formulate future improvements for the institute and even for any specific departments. It was also unclear how scientific programmes (colloquia, seminars, conferences) disseminate research results between clinicians and researchers. Managers reported that other centres across Europe were starting to contact them about ideas for data integration. Data integration was seen as crucial to improve communication between staff and also from staff to patients.

*‘Our information is more accessible and integrated now across research, education and care’*. (Manager, Centre 5 audited)

*‘What information do we have now? What are we missing? How do we obtain it? Ensuring all information that we have is available as a Central intelligence to information, how to fit it under one roof? How can we benchmark ourselves against other Centres and identify new ways of data collection’.* (Manager, Centre 1 unaudited)

### (iii) Improved processes within MDT meetings (procedural change)

The reviewers found that in almost every centre that was peer-reviewed, there were differences in the MDT meetings for admitted patients and outpatients. Although meetings were held every one to two weeks depending on the tumour type, it was unclear if every new patient was being discussed. A general description of the selection criteria to decide which patients were being discussed was not written. However, MDT members knew the criteria. For MDT organisation (outpatient), the specific responsibilities of the physicians in the follow-up period of the patients was not clear, such as who will announce the treatment plan and take care of the patient during his/her follow-up. There were also differences in the procedures of each MDT e.g. urology and melanoma. For the weekly breast cancer meetings, a list of all patients to be discussed was sent in advance to all team members. It also contained information on pathology results and the staging. But for other tumours such a list was not used. Who is responsible for evaluating the execution of the conclusions and advice from the MDT meetings was not clearly described and could not be made clear during the review visit. The procedures describing how the conclusions and advice from the MDT were evaluated and by whom was unclear, but all final decisions of MDT meetings were registered in the patient’s health record.

Staff interviews suggest that the number of meetings between the MDTs has increased and the role of nurses and supportive care personnel in MDTs were clarified. The reviewer’s recommendations brought about more uniformity in MDTs for all tumour types (including in setting up of selection criteria and discussing new research proposals). This led to formally approved rules and guidelines in three of the five centres. Finally, the contact person for each individual patient in each tumour group became more visible. Staff believed that the changes in MDT processes were enabled mainly by the data integration, which in turn facilitated communication and interaction especially between clinicians and researchers. Staff also felt that the improvements in MDT processes were indirectly also related to better training and autonomy of nurses and supportive care personnel.

*‘Integrated data in our centre has been a main reason for improved communication between the multidisciplinary teams especially between researchers and clinicians. Patient care has become more fluid as a result of better communication’.* (Manager, Centre 6, audited)

*‘There has been critical thinking on-going about how to improve palliative care especially the communication to informal carers. How they can be involved in the care process’.* (Clinician, Centre 2, unaudited)

### (iv) Building trust with staff and with public (organisational change)

Building internal trust with staff is connected to data-integration, creation of a quality improvement unit and/or identifying specific staff responsible for total quality improvement of the organisation was strongly recommended by the reviewers to almost all centres. Based on the interviews, this was a perceived change among staff from almost all centres.

*‘People now internally listen to quality advice from quality managers after the external experts came and gave their recommendations. Before there was a lot of scepticism about ideas for quality improvement’.* (Manager, Centre 4, audited)

Accreditation also helped improve the centres credibility with funders (in three audited centres) and made the Centre more reliable with patients. Managers said that other centres had started viewing their centres as role model especially in terms of how they integrate their data and how they are improving the multidisciplinary processes.

Centres use accreditation as a tool for benchmarking the performance of their centres against other centres of similar standing in Europe. This perception was shared among all respondents. The nurses mentioned that they would like the performance of nurses of their Centre to be benchmarked with that of other centres.

*‘There are differences in the education and autonomy of nurses across Europe. We would like to compare our performance with other centres to improve our performance and learn from each other’.* (Nurse, Centre 2, unaudited)

*‘We couldn’t believe that accreditation can have so much power. Before accreditation, we found it difficult to convince decision-makers to make the case for funding. We got more negotiating power since having our audit’*. (Manager, Centre 5, audited)

*‘Other centres now want to come and see our data integration processes and learn from us’.* (Manager, Centre 7, audited)

### Unique view of basic/translational researchers

In centres that had undergone a peer-review, reviewers found that translational research was not structurally and visibly embedded in the centre’s strategy and planning. Some departments have their own strategic plan and it is not clear how this fits in with the institutional level plan. The procedures for allocating resources and funding available for translational research were not clearly defined. The board of directors gave an estimated budget for translational research. The systematic transfer of research results into daily practice though a scientific programme was missing in many of these centres. Based on staff feedback, the centres that had been through the peer-review process have developed and/or refined a five-year plan with clear objectives and actions for translating research into practice. However, it is unclear whether those objectives/actions have yet been put into practice. In addition, indicators for evaluating the actions are largely missing. Budget allocated to research and translational research was unclear in three out of five centres.

During the interviews, almost all researchers reported that they had not experienced any changes from taking part in the accreditation process. They felt that clinical areas gain far more from accreditation than research.

*‘I don’t know if accreditation has brought any visible impact on research teams, the impact is noted more on the clinical side. But certainly communication between research and clinical teams has improved and this has led to more thinking about translational research’.* (Basic/Translational Researcher, Centre 8, audited)

*‘We need more cooperation between researchers and clinicians not only in our Centre but also externally, this can advance translational research. Maybe such collaborations will appear in the near future, the next steps should be applying for joint proposals with other centres to visit other labs and clinics and learn from them’.* (Basic/Translational Researcher Centre 7, audited)

### Data verification with peer-reviewers suggestions to audited centres

**Figure 2.**
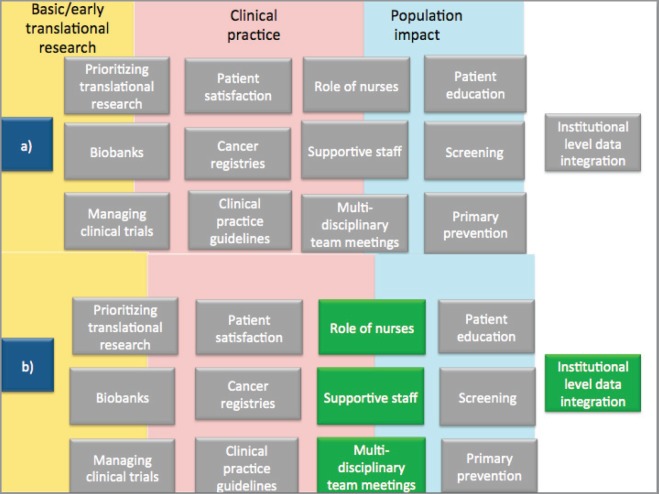
Verification of interview data against peer-reviewers recommendations to audited centres. Note: a) denotes areas where peer-reviewers gave recommendations to audited centres, and b) denotes areas (highlighted in green) where interview participants noted changes attributable to the accreditation programme.

We accessed the accreditation reports of the audited centres to see what recommendations the auditors had given them. The grey boxes in Figure 2 are derived from the recommendations based on the accreditation standards topics as listed in the reports. In Figure 2, part a) summarises 13 areas (presented in grey boxes) where auditors identified the need for changes in audited centres. In the same Figure 2, part b) highlights the four areas (in green boxes) where the interview respondents perceived changes from taking part in the accreditation programme. The remaining nine areas are discussed below:

*Prioritising translational research:* The reviewers emphasised a clear strategic vision for embedding translational research into the mainstream organisational culture. As a next step, it was necessary to identify resources (staff, budget etc.) to make this vision happen. Connected to translational research is the issue of biobanks that is crucial for research.

*Bio-specimen banks (biobanks):* Reviewers pointed out several issues regarding the policy for bio banking and standard operating procedures (SOPs) and concerning collection; storage, registration, quality, and use of biological samples that were not fully implemented at many of the centres across all tumour types.

*Managing clinical trials:* In a few centres, the reviewers found that no full-time staffs were available with clear roles to initiate, conduct, and manage academic clinical trials. Industry sponsored trials were selected and initiated after a review by a separate committee also using good clinical and laboratory practices and the number of patients were also significantly higher in industry-sponsored trials than in academic trials.

*Patient privacy and satisfaction:* In a few centres, reviewers found that facilities for safeguarding patient privacy while sharing information with the patient’s family during treatment, especially at the end of life, and the immediate bereavement period could be much improved. In some cases, relatives could stay overnight with patients but in uncomfortable conditions.

*Cancer registries:* In many centres, the registry is not always used to facilitate future improvement actions in some centres. Waiting times are available but a structural continuous improvement process of monitoring and evaluation by pathology and care pathway leading to define improvement actions of planning and/or organising the care and treatment of departments or the complete hospital was missing.

*Clinical practice guidelines:* Deviations from guidelines are documented in patient’s paper files, which are accessible for the core team. Guidelines were available and accessible on the intranet. The coordination on how guidelines are produced, managed, used, updated was not standardised in each department or within each MDT.

*Patient education:* The reviewers found that in centres, patients and relatives receive oral information about pain management. Written information is only accessible after consultation with a physician since information brochures are stored in the electronic system. Patients are referred to palliative care through the institutional process of requesting internal consultation. In two centres, a written procedure on referring patients to any discipline was not available.

*Screening:* All centres were performing and were responsible for some screening programmes. But in some cases, those programmes only covered a small percentage of the total population. Centres organise, plan, participate, and follow up on these programmes, and report to the funding body on how the money was spent. Centres offer consultation to patients but no full support programme was offered.

*Primary prevention:* The reviewers found that the interaction with General Practitioners is not formally structured in some Centres. There was a lack of written/formal agreements with other institutions e.g. community home care teams or with palliative care/hospice organisations/genetic lab testing were lacking.

## Discussion

The aim of our study was to capture the perceived changes by staff from cancer centres taking part in an accreditation programme. We found Pawson *et al*’s approach [8] to be a useful guide for our categorisation of changes taking place in cancer centres as a result of their participation in an accreditation programme. We found that the statement that dovetailing of types of changes are responsible for everlasting changes [8] was justified. Different types of changes in cancer centres are interlinked. For example, according to staff perceptions, critical thinking about data-integration (strategic change) is a major source for better communication and alignment of processes within MDTs (procedural change). The strategic change also has an impact on the organisational change, for e.g. data integration improved trust with other cancer centres. We did not find any evidence of motivational change in our data. The transtheoretical model of behavioural change [10] suggests that motivational change is a ‘process’ that takes places in different phases, rather than a single event which might explain this. Motivation is needed to move through all these phases of change and seems to form an underlying basis of all change typologies (strategic, administrative, role or procedural).

Our study confirms that accreditation programmes act as an indispensable tool for quality improvements in HCOs. For example, we found that the role and autonomy of nurses improved through this accreditation programme. Also, more supportive care personnel were deemed necessary in centres. In a study of acute care hospitals, the number of infection control nurses increased after the accreditation of medical care services [11]. The nurses that we interviewed suggested that pain management was an important aspect to be improved and also that is where they have perceived improvements. Previous studies suggest that accreditation has positive impact on several clinical outcomes including pain management [12]. Nurses saw the role of accreditation in the improvement of quality care for patients [13] and had an overall positive attitude regarding accreditation [14]. Nurses and managers were the most positive group of stakeholders about accreditation. Clinicians saw the relevance but were largely skeptical about accreditation. They perceive it to be cumbersome and bureaucratic [15]. The basic/translational research group stood out as they did not feel motivated. They felt that accreditation has more potential to improve patient care than research areas of cancer centres. Motivation of organisational members to implement change depends on how they value change [16]. Despite high levels of research resources, few centres have a clear mechanism for integrating research and care e.g. clearer policies/ processes for basic/translational research [5]. A key reason accreditation was preferred by European cancer centres was for benchmarking. This is similar to evidence from a previous Canadian study [17].

By applying Deming’s Plan-Do-Study-Act cycle (PDSA cycle) [18], we can say that the plan and do phases tend to occur before the audit, however, the majority of the do phase and the study and act phases are more likely to occur after the audit. Each PDSA cycle takes time, however, the landscape of oncology research and care is rapidly changing [19]. Unless, change management is part of the organisational culture, planned changes will be prone to external elements [20] that can delay or revoke the implementation of necessary changes.

## Conclusion

We recommend future research to test multiple change theories that capture not just change typologies but also explain how and why change occurs. Accreditation programmes have the potential to bring changes but it is important that they take the differing needs/ expectations of stakeholder groups into account while developing and/or revising their standards. Staff perceptions are one way of interpreting changes. Accreditation agencies as well as participating organisations should invest in tools that more effectively and objectively help track ongoing changes in centres. This will help strengthen the international evidence base on the effectiveness of accreditation.

## Conflicts of interest

The authors’ institutions are members of the Organisation of European Cancer Institute (OECI) and some authors (MS, FB, WvH) are involved in the development of the OECI Accreditation and Designation programme.

## Figures and Tables

**Table 1.** Research Methods presented in consolidated criteria for reporting qualitative studies (COREQ) guidelines checklist: 32-item checklist [6].

**Domain 1: Research team and reflexivity**	**Personal characteristics**	
	1) Which author(s) conducted the interview?	Interviews conducted by AR.
	2) What were the researcher’s credentials?	AR: PhD candidate in health policy/services research; AW: PhD candidate in health policy/services research; FB: MA; MS: MD; FT: PhD candidate in public health; WvH: MD-PhD.
	3) What was their occupation at the time of the study?	AR: PhD candidate; AW: PhD candidate FB: coordinator of the accreditation and designation programme; MS: medical oncologist; FT: PhD candidate; FB: accreditation and designation programme coordinator; WvH: Professor of health services and technology assessment and member of board of directors, The Netherlands Cancer Institute.
	4) Was the researcher male or female?	Five females, one male.
	5) What experience or training did the researcher have?	Experience in conducting qualitative research (AR, AW, FT), expertise in public health/ health services research (all authors), expertise in translational cancer oncology (AR, MS, FT, WvH).
		**Relationship with participants**
	6) Was a relationship established prior to study commencement?	The interviewer did not know the participants before the study.
	7) What did the participants know about the researcher?	At the start of the study, the aim of the research project, as well as the objectives of the study was presented.
	8) What characteristics were reported about the interviewer/facilitator?	Interviewer characteristics were not reported to participants.
**Domain 2: Study design**	**Theoretical framework**	
	9) What methodological orientation was stated to underpin the study?	We used thematic analysis [7]. We used an existing framework developed by Pawson *et al* that captures the typologies of changes in healthcare programmes. We used this framework to note the changes experienced by staff from cancer centres from their participation in the OECI A&D programme. We applied Pawson *et al* theory [8] that deals with ‘different aspects of change in complex organisations by taking a ‘theorydriven review’ underpinned by an understanding of dynamics of social change in complex organisations’. This would suit the complex nature of cancer centres that include research and patient care. This approach argues ‘healthcare interventions are often evaluated by simply presenting (sub-typing) their outcomes but rarely do they provide lessons on how to improve organisations by understanding the types of change that occur’. Pawson *et al*, suggest ‘the need for a ‘theory-driven review’ reinforced by multi-layered types of changes: *Strategic change-* the key change mechanism lies in the better coordination of practices and process by rethinking the entire pathway; *Role change-* this element proposes a dramatic change in which systems and organisational structures remain intact but within which roles and responsibilities are shifted; *Procedural change-* this type of change is at the level of individual practice by increasing the efficiency and effectiveness of procedures. *Motivational change-* includes the practice of offering incentives to encourage key actors to reshape their behaviour. The intended change is behavioural and driven by personal interest rather than peer learning. *Organisational change-* improvements include new produces; a better division of labour, better-cost containment, better information flow, better training. In this approach, lasting system transformation depends on the dovetailing of these types of changes that are interconnected with each other [8]’. We selected this theory to see if these proposed typologies of changes appear in cancer centres and whether the ideology of dovetailing of typologies of changes is relevant to cancer centres.
	**Participant selection**	
	10) How were the participants selected?	We did purposive sampling and invited 32 participants (a researcher, a clinician, a nurse, and a manager from each centre) from eight cancer centres across Europe. These individuals were responsible for gathering data for the audit as well as participating in the audit. This strategy ensured that a diverse group of staff was interviewed from a variety of contexts. five of these centres had been audited. These were located in Finland, Lithuania, Portugal, Spain, and the UK. The remaining three centres were preparing themselves for the audit. They were located in Norway, the UK, and Italy. The centres were either freestanding Comprehensive Cancer Centres (independent entities, not located within a university or a general hospital structure) or they were part of a general university hospital. In the latter case, key facilities e.g. radiology are then not just dedicated to cancer but also to other diseases and specialties. The participating centres agreed that ethical consent was not necessary for this study.
	11) How were the participants approached?	Originally by email.
	12) How many participants were in the study?	Twenty-four: clinicians (five), nurses (six), managers (eight) and basic/translational researchers (five).
	13) How many participants refused to participate or dropped out? Why?	Eight (because of time constraint). However, managers from three centres also gave feedback on behalf of a few clinicians and/or researchers from their Centre (who had handed them their answers as they could not participate in the interview themselves because of lack of time). This indirect feedback from four participants (two researchers and two clinicians) was also taken into account while coding.
	**Setting**	
	14) Where was the data collected?	Telephone interviews were conducted from AR’s office.
	15) Was anyone else present besides the participants and researcher?	No.
	16) What are the important characteristics of the sample?	Diversity of backgrounds and occupation. There were basic/translational researchers, clinicians, nurses, and managers.
	**Data collection**	
	17) Were questions, prompts, guides provided by the author? Was it pilot tested?	The topic guide was to the participants in advance. The interview guide was prepared based on the chapters/topics of the OECI accreditation and designation programme standards.
	18) Were repeat interviews carried out? Details	No repeat interviews.
	19) Did the researcher use audio or visual recording to collect the data?	Yes.
	20) Were field notes made during and/or after the interview or focus group?	Field notes taken during all interviews. Memo made immediately after the interview.
	21) What was the duration of interviews or focus groups?	30–45 minutes.
	22) Was data saturation discussed?	No.
	23) Were transcripts returned to participants for comments and/or correction?	No.
**Domain 3: Analysis and findings**	**Data analysis**	
	24) How many data coders coded the data?	Two authors (AR, AW) created the initial coding tree using five sample interview transcripts.
	25) Did authors provide a description of the coding tree?	No but we show the themes/categories that emerged and the peer-reviewers findings from the cancer centres based on the on-site visit.
	26) Were themes identified in advance or derived from the data?	The themes were derived deductively using Pawson *et al* framework. Our triangulation [9] technique involved two strategies. First, we sent the emergent themes and a brief explanation to all participants. They confirmed that they were able to relate to our findings. As a next step, we analysed auditor’s recommendations for improvement to the five centres. We checked whether these themes related to the themes emerging from staff interviews.
	27) What software, if applicable, was used to manage the data?	NA.
	28) Did participants provide feedback on the findings?	Yes.
		**Reporting**	
	29) Were participant quotations presented to illustrate the themes/ findings? Was each quotation identified?	We present some quotations to illustrate findings.
	30) Was there consistency between the data presented and the findings	The data presented and the findings are consistent.
	31) Were major themes clearly presented in the findings?	We present the most important themes related to the study objectives in the findings.
	32) Is there a description of diverse cases or discussion of minor themes?	Yes.

## References

[1] Alkhenizan A, Shaw C (2011). Impact of accreditation on the quality of healthcare services: a systematic review of the literature. Ann Saudi Med.

[2] Shaw CD (2010). Sustainable healthcare accreditation: messages from Europe in 2009. Int J Qual Health Care.

[3] Kilsdonk MJ (2014). The impact of organisational external peer review on colorectal cancer treatment and survival in the Netherlands. Br J Cancer.

[4] Mount CB (1996). The continuous quality improvement process in dynamic and rapid change. Semin Nurse Manag.

[5] Saghatchian M (2014). Pioneering Quality Assessment in European Cancer Centers: A Data Analysis of the Organization for European Cancer Institutes Accreditation and Designation Program. J Oncol Pract.

[6] Tong A, Sainsbury P, Craig JC (2007). Consolidated criteria for reporting qualitative research (COREQ): a 32-item checklist for interviews and focus groups. Int J Qual Health Care.

[7] Braun V, Clarke V (2006). Using thematic analysis in psychology. Qual Res Psychol.

[8] Pawson R, Greenlagh J, Brennan C (2014). Do reviews of healthcare interventions teach us how to improve healthcare systems?. Soc Sci Med.

[9] Miles MB, Huberman AM, Denzin NK, Lincoln YS (1994). Data management and analysis methods. Handbook of Qualitative Research.

[10] Armitage CJ (2008). Is there utility in the transtheoretical model?. Br J Health Psychol.

[11] Oh HS (2006). National survey of the status of infection surveillance and control programs in acute care hospitals with more than 300 beds in the Republic of Korea. Am J Infect Control.

[12] Frasco PE, Sprung J, Trentman TL (2005). The impact of the joint commission for accreditation of healthcare organizations pain initiative on perioperative opiate consumption and recovery room length of stay. Anesth Analg.

[13] El-Jardali F, Jamal D, Dimassi H (2008). The impact of hospital accreditation on quality of care: Perception of Lebanese nurses. Int J Qual Health Care.

[14] Jaber HM (2014). The impact of accreditation on quality of care: perception of nurses in Saudi Arabia.

[15] Alkhenizan A, Shaw CD (2012). The attitude of health care professionals towards accreditation: A systematic review of the literature. J Family Community Med.

[16] Weiner BJ (2009). A theory of organisational readiness for change. Implement Sci.

[17] Pomey MP, Lemieux-Charles L, Champagne F (2010). Does accreditation stimulate change? A study of the impact of the accreditation process on Canadian healthcare organizations. Implement Sci.

[18] Moen RD, Norman CL (2009). “The History of the PDCA Cycle”,.

[19] Fraher EP, Stinzenberg KP (2013). Building better oncology data systems and workforce models in a rapidly changing health care system. J Oncol Pract.

[20] Drucker PF (1995). Managing in a time of great change.

